# Supplementary material for: Hsa-miR-22-3p inhibits liver cancer cell EMT and cell migration/ invasion by indirectly regulating SPRY2
Source: PLoS One. 2023 Feb 7;18(2):e0281536. doi: 10.1371/journal.pone.0281536 (PMC9904474; doi:10.1371/journal.pone.0281536)
Supplement: S1 Raw images — (PDF) [file pone.0281536.s001.pdf]

Fig 1C

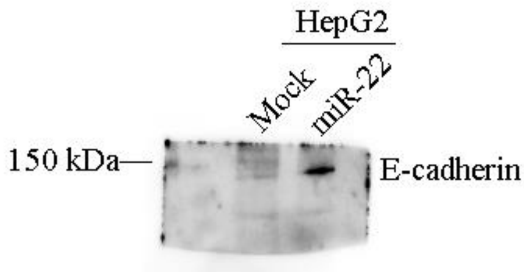

Figure 1C top left panel was generated from this original image.

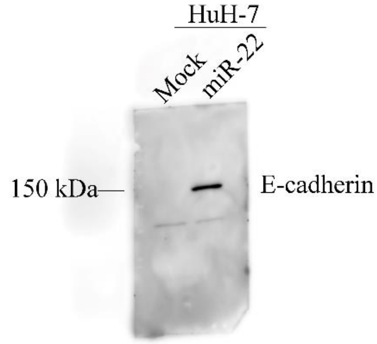

Figure 1C top right panel was generated from this original image.

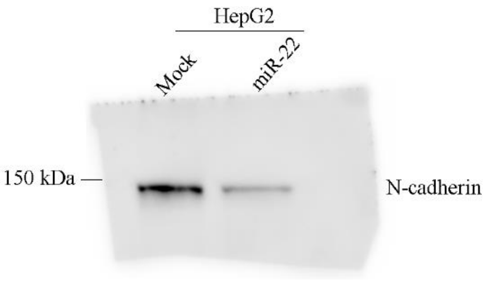

Figure 1C middle left panel was generated from this original image.

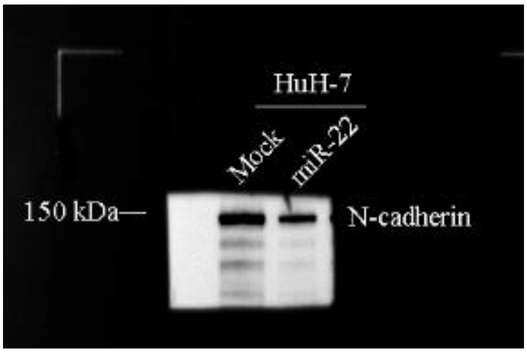

Figure 1C middle right panel was generated from this original image.

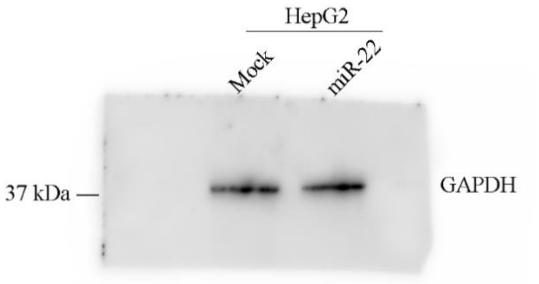

Figure 1C bottom left panel was generated from this original image.

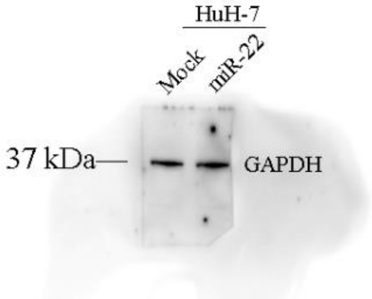

Figure 1C bottom right panel was generated from this original image.

Fig 1H

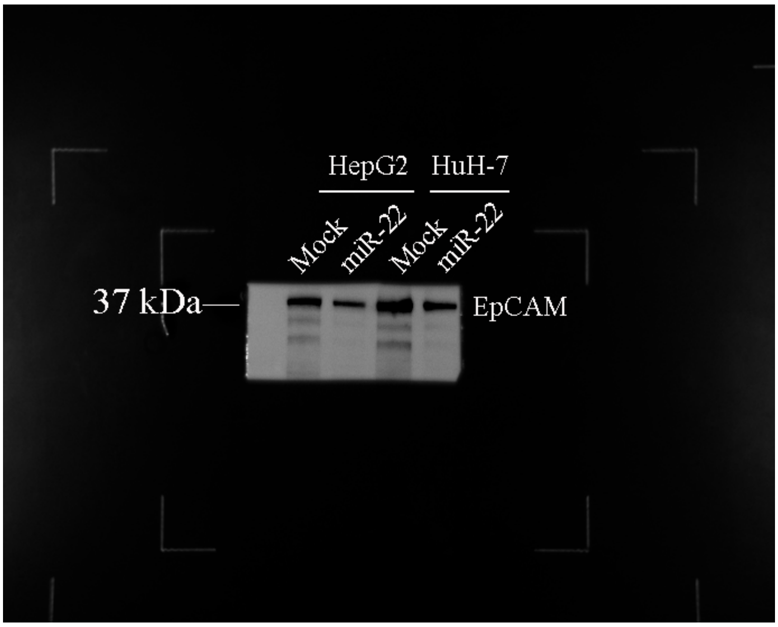

Figure 1H top panel was generated from this original image.

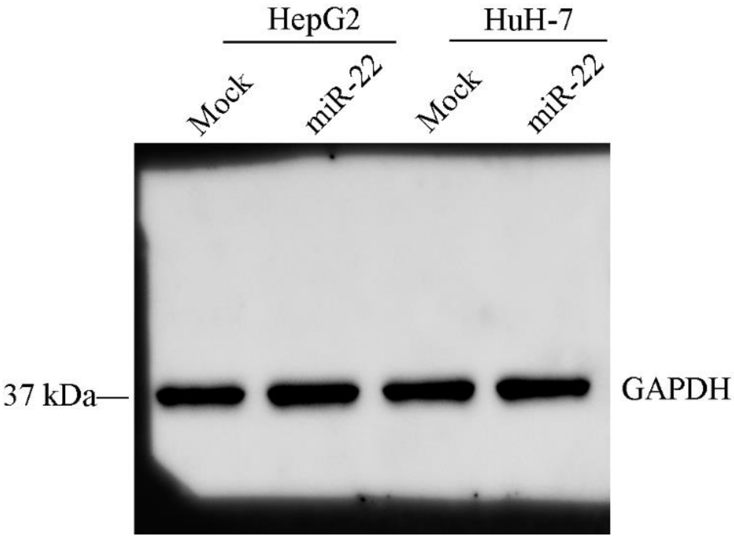

Figure 1H bottom panel was generated from this original image.

Fig 2B

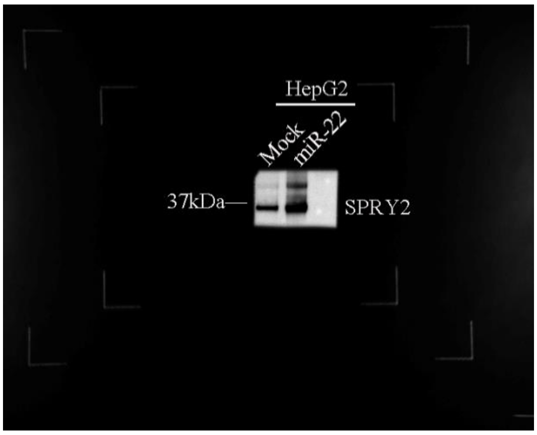

Figure 2B top left panel was generated from this original image.

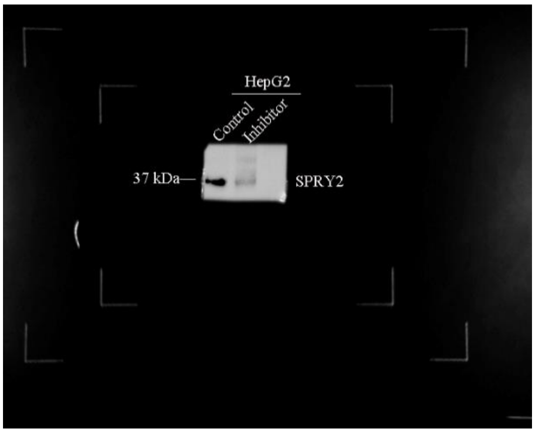

Figure 2B top right panel was generated from this original image.

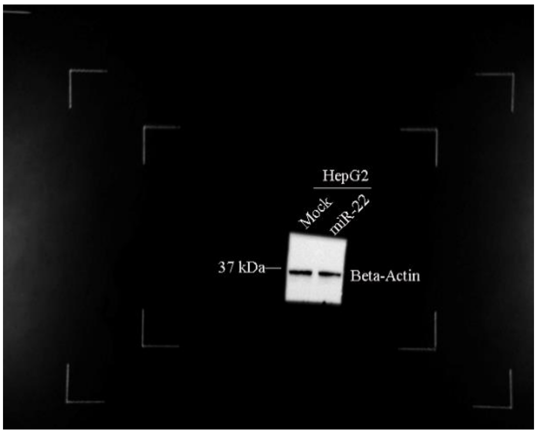

Figure 2B bottom left panel was generated from this original image.

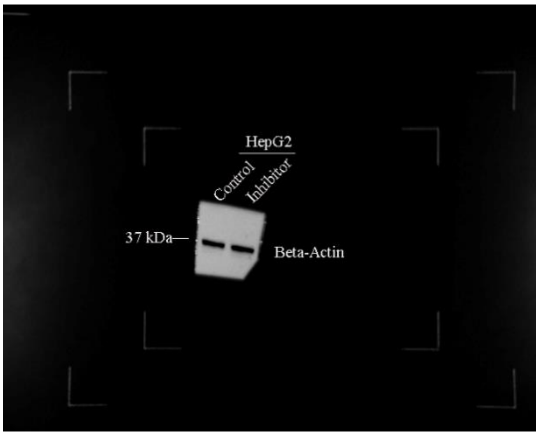

Figure 2B bottom right panel was generated from this original image.

Fig 2C

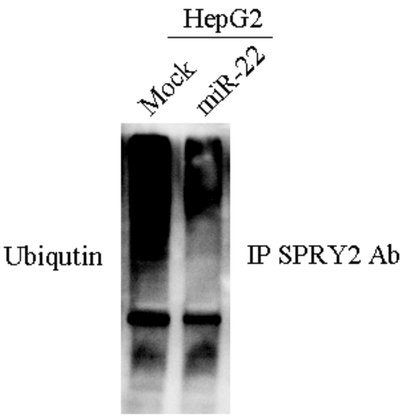

Figure 2C top left panel was generated from this original image.

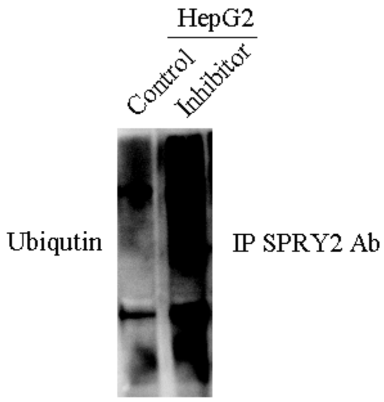

Figure 2C top right panel was generated from this original image.

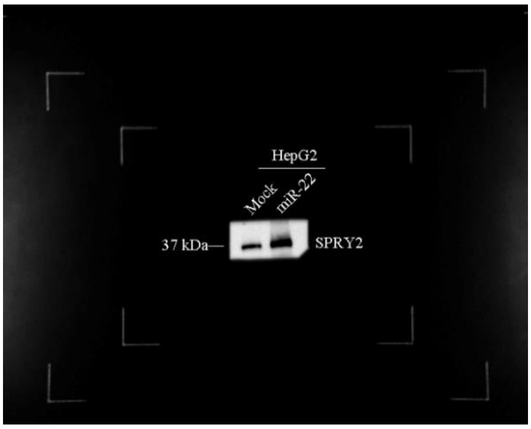

Figure 2C middle left panel was generated from this original image.

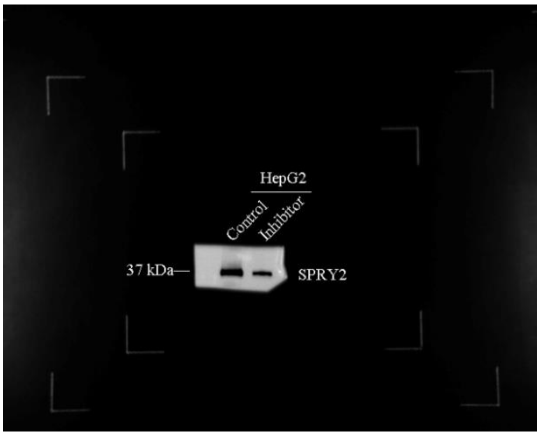

Figure 2C middle right panel was generated from this original image.

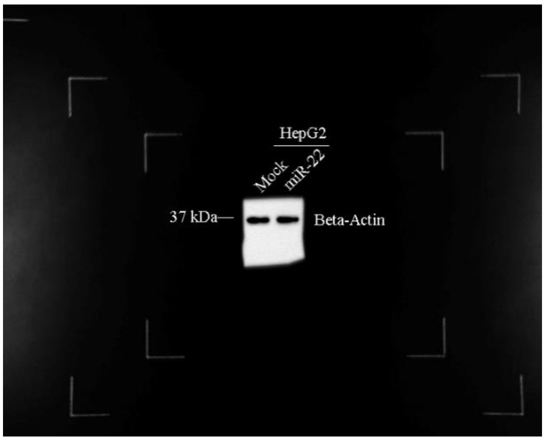

Figure 2C bottom left panel was generated from this original image.

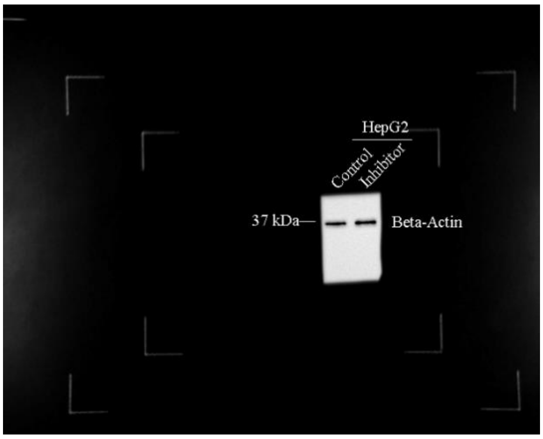

Figure 2C bottom right panel was generated from this original image.

Fig 3A

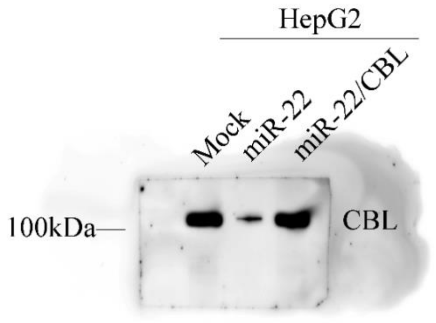

Figure 3A top panel was generated from this original image.

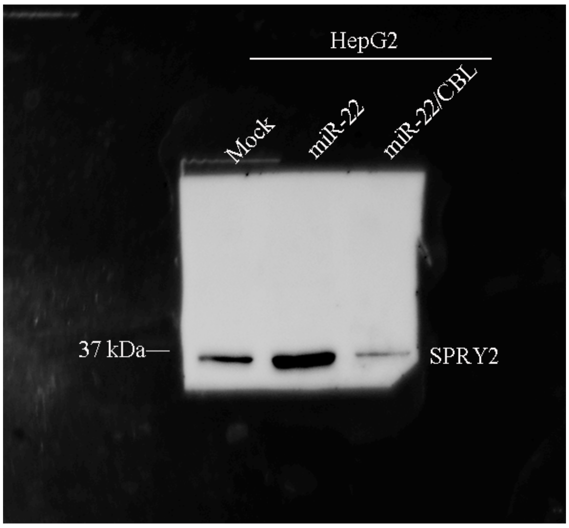

Figure 3A middle panel was generated from this original image.

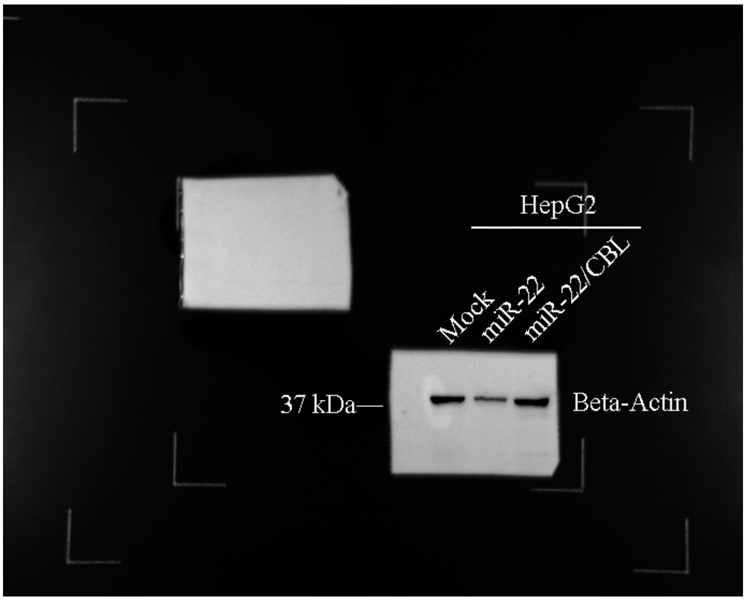

Figure 3A bottom panel was generated from this original image.

Fig 3B

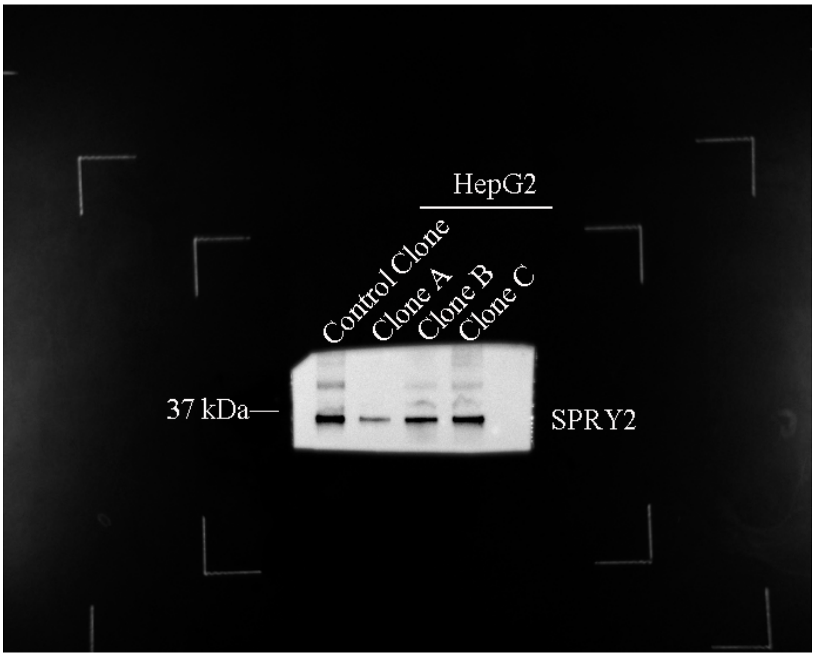

Figure 3B top panel was generated from this original image.

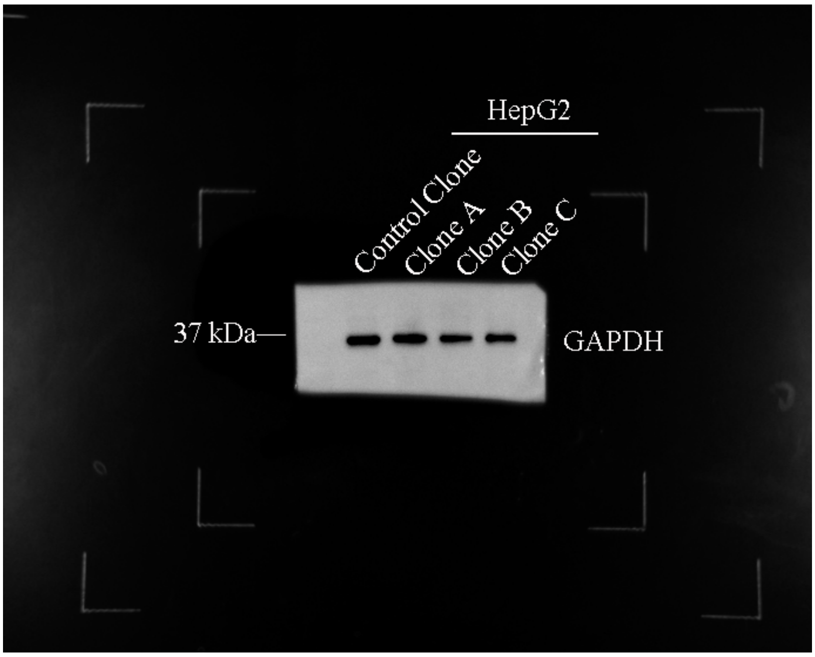

Figure 3B bottom panel was generated from this original image.

Fig 3C

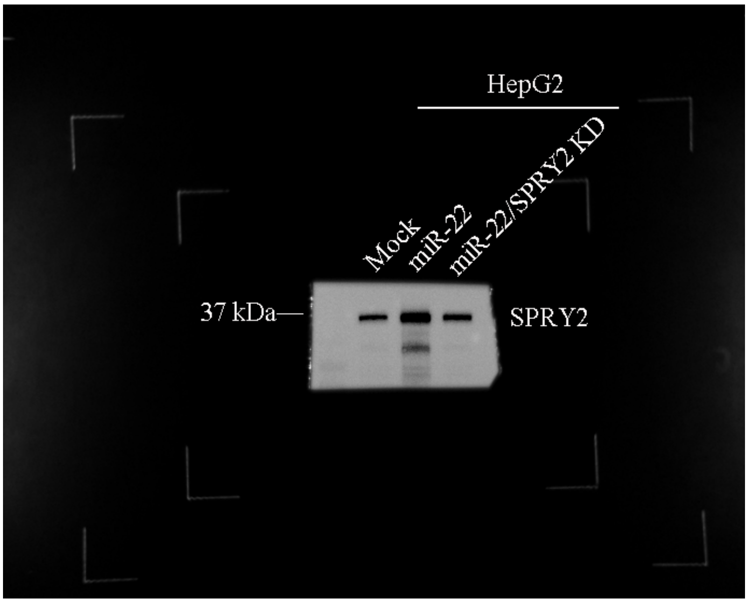

Figure 3C top panel was generated from this original image.

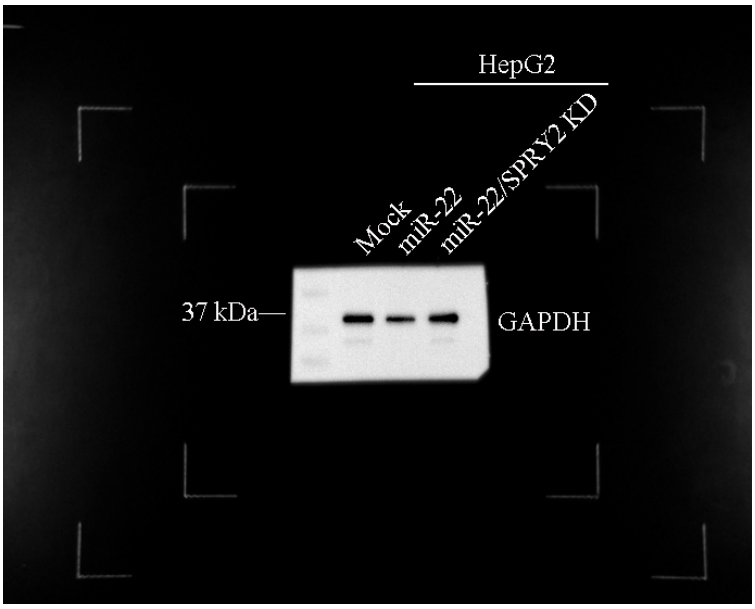

Figure 3C bottom panel was generated from this original image.

Fig 3D

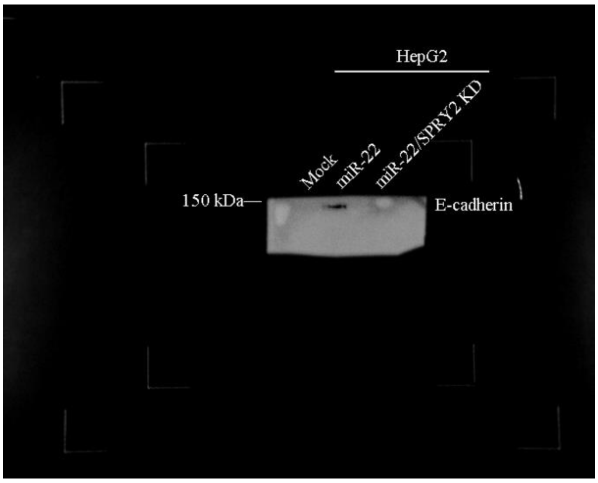

Figure 3D top panel was generated from this original image.

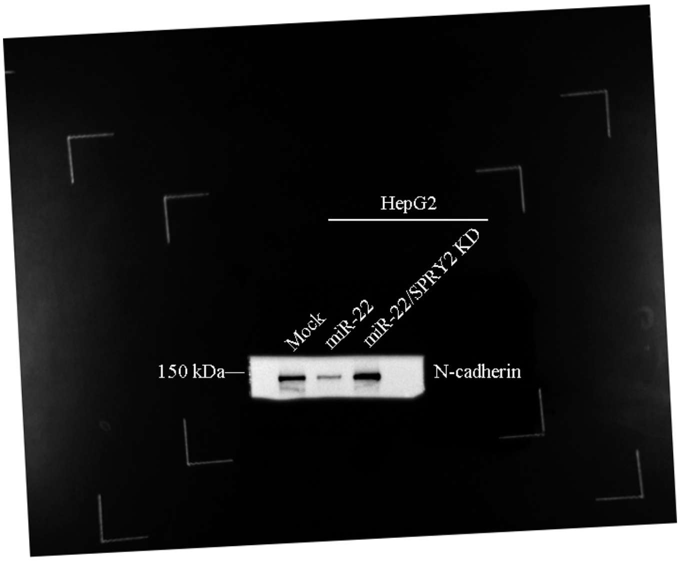

Figure 3D middle panel was generated from this original image.

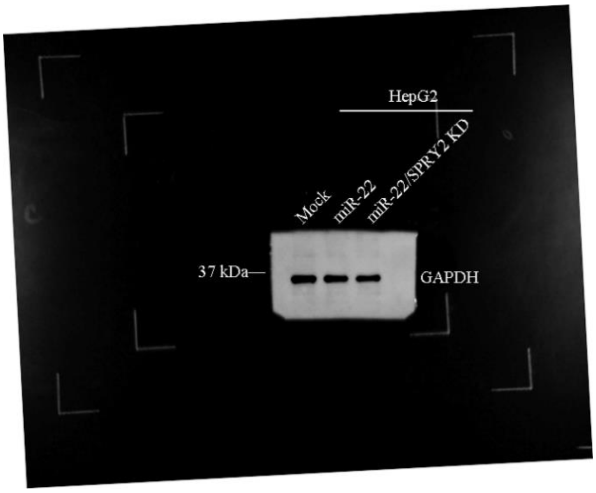

Figure 3D bottom panel was generated from this original image.

Fig 4A

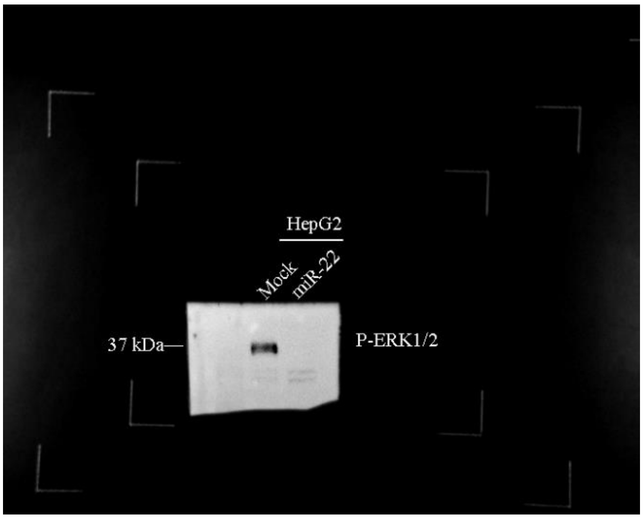

Figure 4A top panel was generated from this original image.

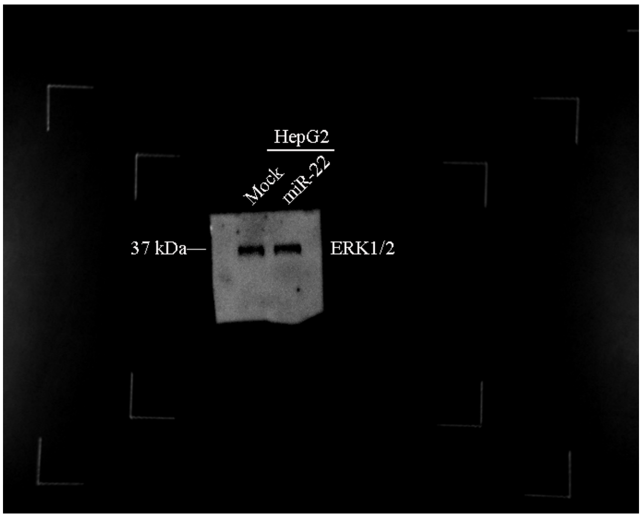

Figure 4A middle panel was generated from this original image.

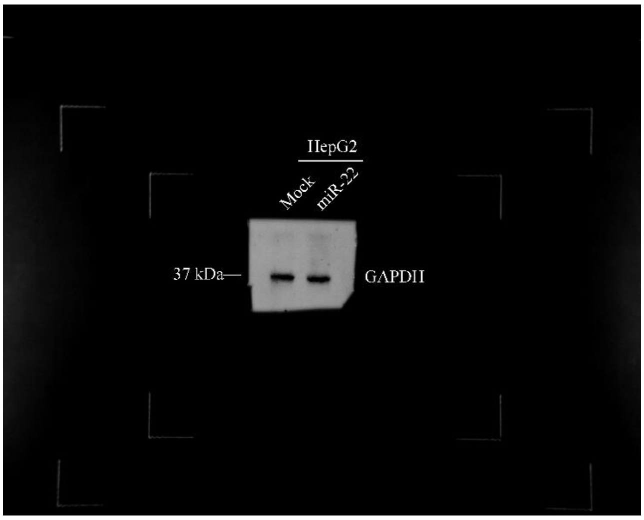

Figure 4A bottom panel was generated from this original image.

Fig 4B

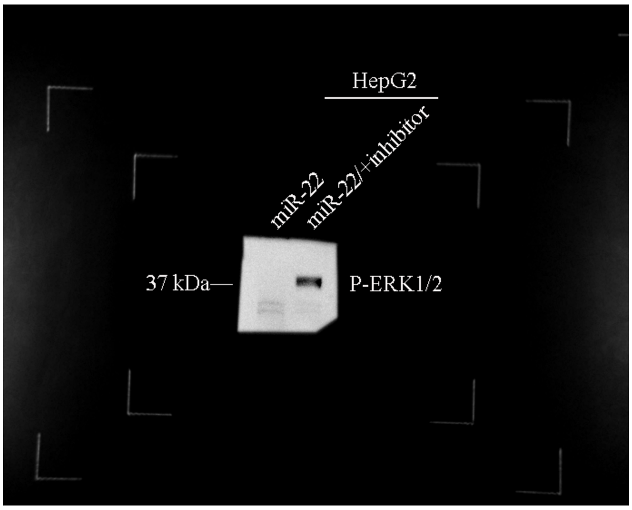

Figure 4B top panel was generated from this original image.

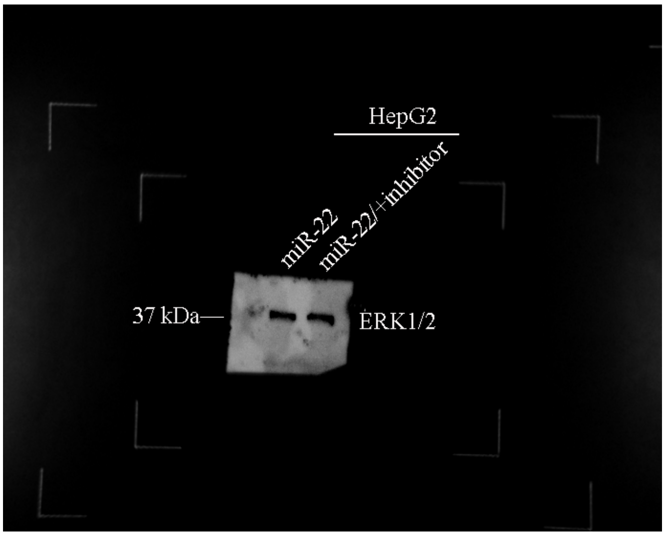

Figure 4B middle panel was generated from this original image.

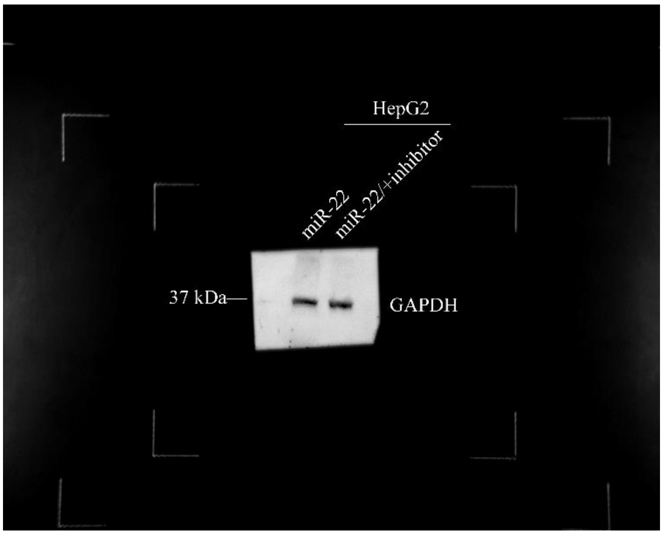

Figure 4B bottom panel was generated from this original image.

Fig 4C

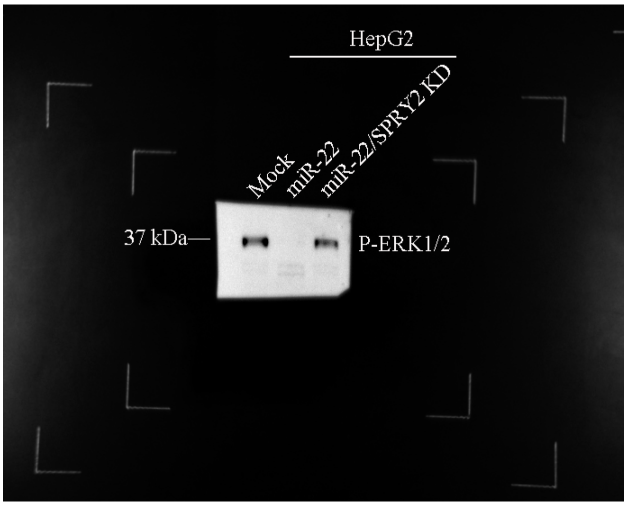

Figure 4C top panel was generated from this original image.

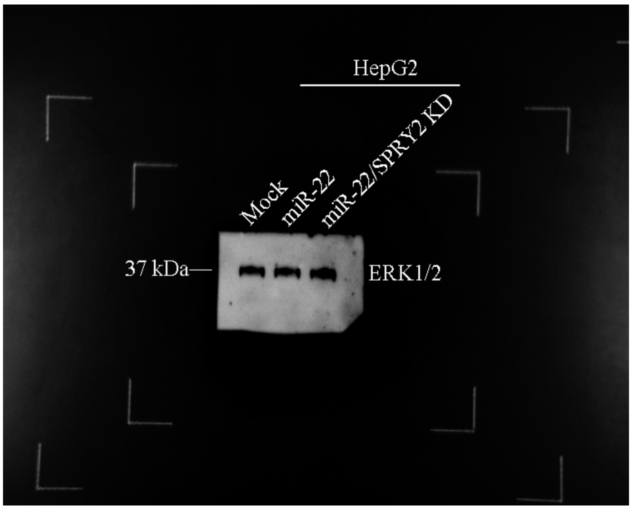

Figure 4C middle panel was generated from this original image.

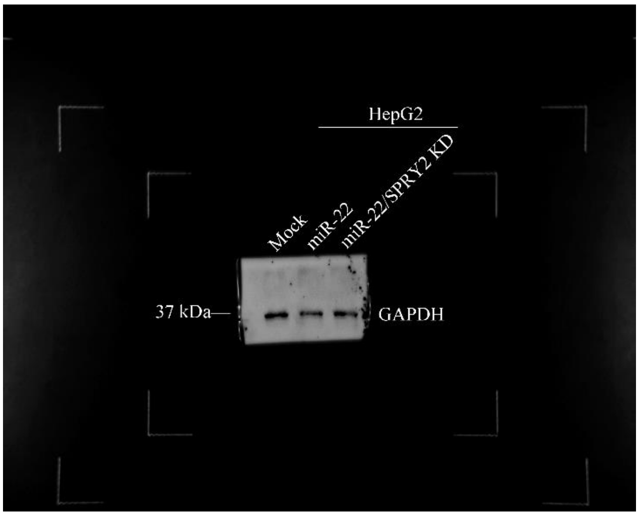

Figure 4C bottom panel was generated from this original image.
